# Association of diet, lifestyle, and chronotype with metabolic health in Ukrainian adults: a cross-sectional study

**DOI:** 10.1038/s41598-024-55715-0

**Published:** 2024-03-01

**Authors:** Mariana Romanenko, Julius Schuster, Liubov Piven, Liudmyla Synieok, Tetyana Dubiley, Liudmyla Bogomaz, Andreas Hahn, Mattea Müller

**Affiliations:** 1 D.F. Chebotarev State Institute of Gerontology, NAMS of Ukraine, Vyshgorodska Str. 67, Kyiv, 04114 Ukraine; 2 Institute of Food Science and Human Nutrition, Leibniz University Hannover, Am Kleinen Felde 30, 30167 Hannover, Germany

**Keywords:** Risk factors, Nutrition disorders, Metabolic syndrome, Nutrition

## Abstract

Morning chronotypes are associated with healthier metabolic profiles and lifestyles compared to evening chronotypes. However, limited research examined the relationship between chronotype, dietary intake, and metabolic health using accurate measures such as food records. This cross-sectional study aimed to investigate the association between chronotype, dietary intake, and metabolic health markers in a cohort of Ukrainian individuals. Chronotypes were determined using the Morningness-Eveningness Questionnaire (MEQ) in 110 healthy to obese individuals (30–75 years) without type 2 diabetes. Dietary intake was derived from weighed seven days food diaries, anthropometrics and blood markers of glucose and lipid metabolism were measured. Morning chronotypes were significantly older and exhibited distinct dietary patterns, including lower intake of fat and animal protein and higher intake of carbohydrates when compared to evening chronotypes (p < 0.01). Higher MEQ scores, reflecting a tendency toward a morning chronotype, were associated with lower BMI, waist circumference, fasting triglycerides, and glucose (p < 0.05). Further, being of morning chronotype predicted better overall metabolic health. These associations remained significant after adjusting for confounders. The findings suggest that morning chronotypes have a different dietary pattern characterized by a more balanced diet and favorable metabolic profile. Synchronizing daily routines with morning preferences could positively influence metabolic health.

## Introduction

Human activity has always been intrinsically connected to the natural rhythms of sleep-wakefulness, which are regulated by the sunlight and day length. However, the rise of technology, globalization and the demands of modern life have led to an increasing number of people working late into the night, far out of sync with their natural physiological rhythms. This may lead to chronic misalignment between internal circadian rhythms and local time, which is further exacerbated by artificial lighting that enables activity at any time of the day. The adverse metabolic consequences of this mismatch have been well-studied in shift workers^1,2^. Chronotypes, which reflect inter-individual differences in synchronization with the light–dark cycle, categorize individuals as either "larks" or "owls." Morning types, or larks, generally tend to go to bed earlier than evening types on both workdays and during their free days^3,4^. Distinct chronotypes exhibit metabolic profiles that differ in regard to blood glucose, glycated hemoglobin, triglycerides, and low-density lipoprotein cholesterol (LDL-C) concentrations. Notably, individuals with an evening chronotype have an increased risk for cardiometabolic diseases, as highlighted in a recent review^5^. Lifestyle factors such as dietary patterns, meal schedules, physical activity, and sleep timing—rather than genetic predisposition—influence the energy metabolism in evening chronotypes^6^. In fact, evening-type individuals tend to be less physically active and engage in poor eating behaviors, such as emotional and stress-related eating^6,7^. Evening chronotypes are prone to unhealthy eating patterns (i.e., delaying meal timing, skipping breakfast, irregular food intake) and lower overall diet quality, as evidenced by more frequent consumption of fast food, energy drinks, and alcohol^8,9^. This observation is corroborated by their low adherence to healthy dietary patterns^10^.

Despite numerous studies investigating the nutritional aspects of different chronotypes^8,9^, there is a scarcity of research focusing on the consumption of major food groups and detailed nutrient intake. Furthermore, the findings from these studies have yielded conflicting results, particularly regarding specific food groups. Variations in the consumption of grains, fruit, and fish between evening and morning chronotypes have been reported inconsistently across different studies^8^. Regarding macronutrients, most studies did not find significant differences in total energy and fat intake between chronotypes, although protein intake was either similar to or lower in evening chronotypes. Total carbohydrate intake was similar in chronotypes in half of the previous conducted studies, while others reported both lower or higher carbohydrate intake in the evening compared to morning chronotypes^8^. These discrepancies can be attributed to variations in methodological approaches, as most studies relied on food frequency questionnaires (FFQs) and other questionnaires to collect data on food groups and nutrient intake. Only a limited number of studies utilized more accurate methods, such as weighed food records.

Therefore, the objective of the cross-sectional study was to investigate the association between chronotypes, dietary intake (i.e., macronutrient and food groups), and blood parameters of glucose and lipid metabolism in individuals with a wide range of metabolic phenotypes.

## Results

A total of 110 subjects were included in the final analytical sample (Supplementary Fig. S1), of whom 44.5% were defined as morning type, 47.3% belonged to neither evening nor morning (intermediate) type, and 8.2% were defined as evening type. For further analysis considering the small number of subjects belonging to the evening type the sample was dichotomized around the median Morningness-Eveningness Questionnaire (MEQ) score into morning chronotype (≥ 58 points) and evening chronotype (< 58) groups. Subjects in the morning group were predominantly (87.5%) morning chronotype with a mean MEQ score of 63.5 ± 3.9, and subjects in the evening group were predominantly (88.3%) intermediate type with a mean MEQ score of 48.7 ± 7.0 (p < 0.001 for mean MEQ score). Thus, the between-group comparison reflects the difference between morning and intermediate types rather than between morning and evening types of original MEQ.

Morning chronotypes were significantly older (p = 0.001), but the proportion of males and females in both groups did not differ (Table 1). Both chronotypes were comparable in BMI, waist circumference (WC), lipid profiles, and glucose metabolism parameters. At the same time, morning types had a significantly higher waist-to-hip ratio (WHR), p = 0.034. The number of subjects with hypertension and metabolic syndrome (MetS) was similar in both groups.

**Table 1:** Metabolic profile, sleep-related, and lifestyle variables of chronotypes.

	Total (N = 110)	Morning chronotype (N = 56)	Evening chronotype (N = 54)	P-value
Age (years)	56.2 ± 15.8	61.1 ± 15.4	51.0 ± 14.6	**0.001**
Sex (% of females)	79 (71.8)	41 (73.2)	38 (70.4)	0.833
Secondary school	2 (1.8)	1 (1.8)	1(1.9)	0.904
Secondary vocational education	21 (19.1)	10 (17.9)	11(20.4)	
Higher education	87 (79.1)	45 (80.4)	42(77.8)	
Employed	72 (65.5)	33 (58.9)	39 (72.2)	0.164
Retired or unemployed	38 (34.5)	23 (41.1)	15 (27.8)	
BMI (kg/m^2^)	27.0 ± 5.5	27.0 ± 5.2	27.0 ± 5.7	0.990
Waist (cm)	87.0 ± 13.1	87.3 ± 12.0	86.7 ± 14.2	0.828
WHR	0.8 ± 0.1	0.9 ± 0.1	0.8 ± 0.1	**0.034**
Self-reported hypertension (%)	41 (37.3)	26 (46.4)	15 (27.8)	0.050
MetS (%)	21 (19.1)	12 (21.4)	9 (16.7)	0.630
Total cholesterol (mmol/L)	5.7 ± 1.1	5.9 ± 1.1	5.6 ± 1.0	0.111
Triglycerides (mmol/L)	1.1 ± 0.4	1.1 ± 0.5	1.1 ± 0.4	0.551
HDL-C (mmol/L)	1.7 (1.5–1.8)	1.7 (1.6–1.8)	1.7 (1.4–1.8)	0.367
LDL-C (mmol/L)	3.6 ± 1.0	3.7 ± 1.0	3.5 ± 1.0	0.201
Glucose (mmol/L)	5.2 ± 0.6	5.1 ± 0.6	5.3 ± 0.6	0.271
Insulin (μIU/mL)	12.8 ± 9.5	12.0 ± 9.9	13.6 ± 9.0	0.173
HOMA-IR	2.9 ± 2.1	2.7 ± 2.2	3.2 ± 2.1	0.130
Sleep				
Sleep onset on workdays (time)	23.6 ± 1.1	23.0 ± 0.8	24.2 ± 1.0	**< 0.001**
Sleep duration on workdays (h)	7.4 ± 1.1	7.6 ± 1.1	7.2 ± 1.2	**0.045**
Sleep onset on free days (time)	23.8 ± 1.0	23.2 ± 0.8	24.4 ± 1.0	**< 0.001**
Sleep duration on free days (h)	8.0 ± 1.2	8.0 ± 1.3	8.1 ± 1.2	0.522
MSFsc (time)	3.6 ± 1.0	3.1 ± 0.7	4.1 ± 0.9	**< 0.001**
Social jet lag (h)	0.5 (0.0–0.8)	0.3 (0.0–0.7)	0.5 (0.2–1.2)	**0.036**
PSQI score	6.0 ± 2.7	5.8 ± 2.5	6.2 ± 2.8	0.371
PSQI score > 5 (%)	55 (50.0)	27 (48.2)	28 (51.9)	0.849
Physical activity				
IPAQ total MET, minutes per week	4076.0 ± 2758.3	4226.9 ± 2797.8	3919.5 ± 2734.1	0.900
Sitting, hours per day	5.1 (4.0–7.0)	4.8 (3.0–6.1)	6.0 (4.0–8.0)	**0.016**
Smoking				
Current smokers (%)	10 (9.1)	4 (7.1)	6 (11.1)	0.523

Data are mean ± SD or median (IQR). Group differences were assessed using a t-test for means, Mann–Whitney U-test for medians, and Fisher's exact test for categorical variables.

Significant values are in bold.

*BMI* Body Mass Index, *WHR* waist-to-hip ratio, *MetS* Metabolic Syndrome, *HDL-C* high-density lipoprotein cholesterol, *LDL-C* low-density lipoprotein cholesterol, *HOMA-IR* homeostatic model assessment of insulin resistance, *MSFsc* the midpoint of sleep on free days corrected for sleep debt over the working week, *PSQI* Pittsburg Sleep Quality Index, *IPAQ* International Physical Activity Questionnaire, *MET* metabolic equivalent of task.

Sleep onset time was significantly lower in morning chronotypes both on work days and free days (p < 0.001), which was consistent with an earlier time of MSFsc, a sleep debt corrected mid-sleep point (p < 0.001, Table 1). Sleep duration in morning-type subjects was slightly longer on working days (p = 0.045). There was no difference on days off. Social jetlag (SJL) was lower in morning chronotypes compared to evening chronotypes (p = 0.036). Sleep quality, as measured by the Pittsburgh Sleep Quality Index (PSQI) total score, and the number of subjects with poor overall sleep quality (PSQI score greater than 5) was the same in both chronotype groups. Both groups had similar levels of physical activity according to total Metabolic Equivalent of Tasks (MET) minutes per week assessed by International Physical Activity Questionnaire (IPAQ). However, the number of hours spent sitting was lower in the morning types (p = 0.016).

### Dietary intake differs between chronotypes

As shown in Table 2, morning chronotypes consumed less total and animal protein (p = 0.034 and p = 0.001, respectively), and more plant protein (p = 0.016). Further, morning chronotypes had a lower intake of fat (p = 0.001) and especially animal fat (p = 0.005). Total carbohydrate and starch intakes were higher in morning chronotypes compared to evening chronotypes (p = 0.001 and p = 0.010, respectively). Similar, the morning chronotype also had a higher percentage of energy intake derived from carbohydrates (p < 0.001) and consumed more fiber (p = 0.009). Regarding food group consumption, we observed a lower intake of meat and eggs (p = 0.013), and a higher bread intake (p = 0.030) in morning chronotypes as compared to evening chronotypes.

**Table 2:** Dietary intake of chronotypes.

	Total (N = 110)	Morning chronotype (N = 56)	Evening chronotype (N = 54)	P-value
Nutrient intake^1^				
Total protein (g/day)	74.8 ± 12.1	72.1 ± 9.8	77.4 ± 13.7	**0.034**
% E total protein	15.3 ± 2.6	14.8 ± 2.0	15.8 ± 2.9	0.055
Animal protein (g/day)	44.8 ± 14.8	40.2 ± 12.5	49.3 ± 15.5	**0.001**
Plant protein (g/day)	30.0 ± 7.6	31.9 ± 8.8	28.2 ± 5.8	**0.016**
Total fat (g/day)	85.5 ± 13.0	81.2 ± 11.9	89.7 ± 12.7	**0.001**
% E total fat	39.1 ± 5.9	37.2 ± 5.4	41.1 ± 5.8	**0.001**
Animal fat (g/day)	53.8 ± 13.9	49.9 ± 13.5	57.6 ± 13.4	**0.005**
Plant fat (g/day)	31.3 ± 10.4	30.8 ± 10.2	31.7 ± 10.6	0.658
Carbohydrate (g/day)	220.6 ± 35.5	232.7 ± 31.6	208.8 ± 35.5	**0.001**
% E carbohydrate	45.1 ± 7.2	47.7 ± 6.4	42.7 ± 7.2	**< 0.001**
Sugar (g/day)	80.3 ± 25.3	82.4 ± 23.8	78.3 ± 26.7	0.411
Starch (g/day)	116.3 ± 34.4	125.1 ± 30.8	107.7 ± 35.9	**0.010**
Fiber (g/day)	18.4 ± 5.7	19.9 ± 6.4	16.9 ± 4.5	**0.009**
Total energy (kcal/day)	1968.0 ± 464.1	1931.9 ± 444.3	2003.4 ± 484.5	0.442
Food group intake (g/day)				
Total dairy	187.6 ± 135.3	171.3 ± 134.8	203.6 ± 135.1	0.232
Milk/Kefir/Yogurt	89.9 (41.5–171.4)	70.7 (30.6–162.0)	94.0 (45.4–200.1)	0.241
Cheese	59.6 (31.4–88.6)	53.6 (21.5–82.3)	64.3 (40.0–103.3)	0.058
Total meat/eggs	127.1 ± 69.0	109.9 ± 57.0	144.0 ± 76.9	**0.013**
Red meat	22.3 (4.3–47.3)	20.7 (3.4–43.1)	30.0 (5.7–63.0)	0.188
Poultry	32.9 (14.3–62.9)	33.5 (17.1–62.3)	30.3 (12.1–64.0)	0.949
Processed meat	4.3 (0.0–20.0)	0.0 (0.0–18.3)	8.6 (0.0–21.4)	0.172
Egg	30.6 (15.9–43.8)	28.6 (10.4–40.6)	33.3 (19.7–58.6)	0.123
Fish/seafood	24.3 (7.5–44.6)	18.6 (7.1–43.6)	27.4 (10.0–49.7)	0.338
Starchy food	239.4 ± 112.2	258.5 ± 112.2	220.7 ± 110.1	0.090
Cereals/pasta, dry weight	66.9 ± 48.1	68.7 ± 46.3	65.1 ± 50.1	0.704
Bread	74.5 ± 46.0	84.5 ± 45.8	64.7 ± 44.5	**0.030**
Potato	85.0 ± 62.6	93.3 ± 68.4	76.8 ± 55.8	0.187
Vegetables/legumes	225.4 (146.0–335.9)	245.8 (143.8–364.3)	211.9 (155.1–315.1)	0.639
Vegetables	214.4 (142.6–330.9)	228.7 (136.7–354.6)	201.9 (146.0–313.0)	0.704
Legumes	0.0 (0.0–10.0)	0.7 (0.0–16.6)	0.0 (0.0–6.4)	0.237
Berries/fruit	132.0 (55.8–233.0)	122.1 (44.0–230.0)	144.9 (71.3–255.8)	0.302
Nuts/seeds/peanuts	2.1 (0.0–11.0)	1.9 (0.0–10.1)	2.9 (0.0–13.3)	0.552
Confectionery/sweet bakery products	59.9 ± 44.9	65.3 ± 51.9	54.7 ± 36.5	0.240
Honey and sugar	21.0 ± 14.7	21.3 ± 14.2	20.6 ± 15.2	0.796

Data are mean ± SD or median (IQR). Group differences were assessed using a t-test for means, Mann–Whitney U-test for medians, and Fisher's exact test for categorical variables.

Significant values are in bold.

^1^Nutrient intake was energy-adjusted by the residual method, macronutrients are additionally presented as a % of energy. E, energy.

Morning chronotypes had their last eating occasion earlier in the evening compared to the evening chronotypes (p = 0.020), while the time of first eating occasion was similar (Table 3). Other eating times did not differ between the two chronotype groups.

**Table 3:** Temporal eating pattern of chronotypes.

	Total (N = 110)	Morning chronotype (N = 56)	Evening chronotype (N = 54)	P-value
First eating occasion (time)	8.0 (7.5–9.0)	8.0 (7.5–9.0)	8.5 (7.5–9.6)	0.066
Last eating occasion (time)	20.2 ± 1.5	19.8 ± 1.3	20.5 ± 1.7	**0.020**
Fasting window (h)	12.3 ± 2.2	12.3 ± 2.1	12.2 ± 2.4	0.746
Skipping breakfast (%)	7 (6.4)	1 (1.8)	6 (11.1)	0.058
Regular eating time (%)	67 (60.9)	36 (64.3)	31 (57.4)	0.558

Data are mean ± SD or median (IQR). Group differences were assessed using a t-test for means, Mann–Whitney U-test for medians, and Fisher's exact test for categorical variables.

Significant values are in bold.

**Figure 1:**
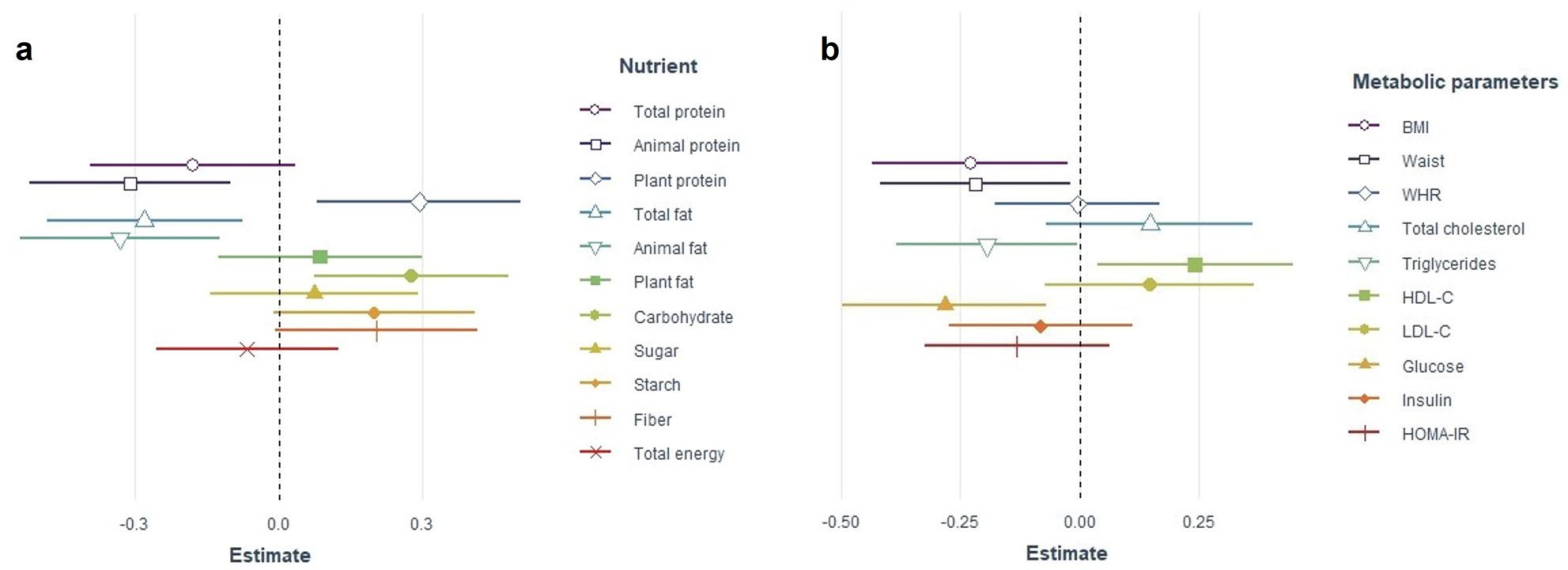
Association of chronotype (MEQ score) with nutrient intake and metabolic parameters. (**a**) Nutrient intake. Mean daily nutrient intake (g/day) as a dependent variable. Nutrient intake was energy-adjusted by the residual method. The overall model for plant protein intake is not significant. (**b**) Metabolic parameters. Metabolic parameter as a dependent variable. In all models the association is calculated for every 10-point change in the MEQ score. Point shapes are standardized beta-coefficient (estimate) and horizontal lines are 95% CI of beta-coefficients. All models are controlled for age, sex, and physical activity. Models for metabolic parameters are additionally controlled for BMI, except models for BMI, waist circumference, and WHR. *MEQ* Morningness-Eveningness Questionnaire, *CI* confidence interval, *BMI* body mass index, *WHR* waist-to-hip ratio, *HDL-C* high-density lipoprotein cholesterol, *LDL-C* low-density lipoprotein cholesterol, *HOMA-IR* homeostatic model assessment of insulin resistance.

Next, we constructed linear regression models to investigate the associations of MEQ score and nutrient intake independent of confounding factors such as age, sex, and physical activity (Fig. 1a, Supplementary Table S1). Higher MEQ scores indicate a tendency towards a morning chronotype, and lower MEQ scores indicate a tendency towards the evening chronotype. After adjustment for age, sex, and physical activity, a higher MEQ score was significantly associated with lower animal protein (p = 0.004), fat, especially animal fat consumption (p = 0.008 and p = 0.002, respectively), and a lower percentage of daily calories from fat (p = 0.007). A higher MEQ score was also associated with higher carbohydrate intake and the percentage of daily calories from carbohydrates (p = 0.008 and p = 0.005, respectively). MEQ scores were not associated with total energy, total protein, plant protein, starch, and fiber intake in linear regression models. Due to skewness, association with food groups and MEQ were investigated using Spearman’s partial correlation showing that MEQ scores were significantly but weakly correlated with cheese (r = − 0.21, p = 0.042), processed meat (r = − 0.20, p = 0.047), and egg intake (r = − 0.21, p = 0.041, Supplementary Table S2).

### Association of chronotype with metabolic parameters

Next, we examined the association between the MEQ scores with metabolic risk factors in linear regression models adjusted for age, sex, physical activity, and BMI (Supplementary Table S3). After adjustment, higher MEQ scores were associated with lower WC, BMI, serum triglycerides, glucose, and higher high-density lipoprotein cholesterol (HDL-C) (Fig. 1b). Age was a significant confounder for all metabolic parameters. Nevertheless, age per se was accompanied by weak changes in MEQ score, implying a shift to a more morning type (std. B (SE) = 0.27 (0.09), std. 95% CI 0.08–0.45, p = 0.005, R^2^ adjusted = 0.063). The relationship between overall metabolic health status and chronotype was estimated by logistic regression models. After adjustment for age, sex, and physical activity, each 10-point increase in MEQ score decreased the risk of being metabolically unhealthy (OR 0.451, 95% CI 0.255–0.798; p = 0.006). MEQ score remained a weak but significant independent predictor of metabolic health along with age and BMI after further controlling for BMI (OR 0.487, 95% CI 0.239–0.990; p = 0.047).

## Discussion

In this Ukrainian cohort, subjects belonging to the morning chronotype exhibited distinct dietary patterns characterized by a more balanced diet and an earlier timing of the last eating occasion. Specifically, morning chronotypes reported lower fat intake, higher carbohydrate consumption, and a reduced intake of animal protein-rich foods. Despite being older in age, morning individuals spent less time in sedentary activities and demonstrated a greater alignment with their biological clock, as reflected by smaller SJL. Moreover, being a morning chronotype was associated with improved metabolic parameters and predicted better overall metabolic health. Importantly, these effects remained significant even after accounting for confounding factors such as sex, age, physical activity, and BMI.

Consistent with previous findings, morning subjects in this study consumed less fat and more carbohydrate compared to evening subjects^11–13^. Interestingly, we observed a lower consumption of animal protein in morning chronotypes, which has not been investigated in previous studies. Linear regression between the continuous MEQ score and nutrient intake models adjusted for confounders confirm the observed group differences; lower consumption of fat, animal protein were consistently associated with higher MEQ scores, i.e., morning chronotypes. The results of the present study partially align with Mota et al., who reported a decrease in total protein intake in medical residents with higher MEQ scores^14^. The lower intakes of fat and animal protein in morning subjects may be attributed to a lower overall intake of animal foods, which is supported by a decreased consumption of processed meat, eggs, and cheese in morning vs. evening chronotypes. A lower intake of meat was also found in morning-type young Japanese women as compared to those of evening-type, although the percentage of energy from protein showed an opposite trend^13^. Regarding fish intake, we did not observe any differences between chronotypes, which is consistent with the findings by Sato-Mito et al.^13^ and is contrary to studies by Kanerva et al.^12^. This discrepancy could be due to the low consumption of fish and seafood the present study population as compared to national consumption rates^15^. In addition, higher carbohydrate, fiber and bread intake were observed in morning chronotypes, yet these differences were maintained only for carbohydrate in linear regression models after adjustment for confounders. As shown in previous studies, MEQ scores were positively associated with dietary fiber intake in Finnish^12^ and Mexican^11^ populations, whereas no association was found in the Spanish population^11^. Furthermore, in a subgroup of the same Finnish population, a more detailed analysis showed a positive association with fiber intake only for morning meals, but not for the whole day^16^. Regarding cereal intake, previous results have been somewhat contradictory^8^. For example, higher intakes of cereals and whole grains were positively associated with MEQ scores in several studies^6,12^. However, another study found no association with cereals, bread, and pasta^14^. Previous studies analyzing healthy diet indices showed that morning chronotypes are more adherent to plant-based dietary patten such as Mediterranean diet^17,18^ and have higher healthy plant-based diet index scores that partially corresponds to our results^19^. The observed discrepancies between nutrient and food group intake may be related to geographic (particularly latitude) and therefore climatic and cultural influences on dietary patterns, limiting the comparability of existing data. On the other hand, these discrepancies highlight the importance of assessing overall dietary quality. Combining food diaries with healthy diet indices would provide a more comprehensive assessment of the association between chronotype and diet, as food records offer valuable quantitative data, while adherence to a healthy dietary pattern may have stronger predictive potential than the consumption of individual foods^18^.

We did not observe differences in metabolic profiles in the between-group comparison of the two chronotypes. However, these differences became apparent upon further multivariate regression analysis adjusted for age, sex, and physical activity, as was observed by others^20,21^. Consistent with previous findings, regression models in the present study found a relationship between higher MEQ scores and lower BMI^6,18,22^, triglycerides^11^, and blood glucose^22^. No association was found between chronotype and hypertension. Partially in line with our results, a recent systematic review found that evening chronotypes exhibited higher concentrations of blood glucose, glycated hemoglobin, triglycerides, and LDL-C, while no significant differences were observed for anthropometric measurements, arterial blood pressure, insulin, the homeostatic model assessment of insulin resistance (HOMA-IR), total cholesterol, and HDL-C^5^. In contrast to others, we observed lower WC and higher HDL-C associated with morning type and no difference in LDL-C. The reported association between a more morning chronotype and lower odds of being metabolically unhealthy in the present study confirms recent findings of the positive association between the evening chronotype and MetS^6,11,20^.

The underlying mechanisms for the metabolic effect of diurnal dietary differences in distinct chronotypes are based on the circadian physiology of metabolism and the zeitgeber effect of food^23,24^. For example, diet-induced thermogenesis and insulin sensitivity vary throughout the day with their impairment in the evening^25,26^, late dinner time causes lower lipolysis and dietary fatty acid oxidation in the postprandial period^27^ meaning a greater metabolic load of the late meal. Distinct eating patterns (i.e., meal timing and regularity, meal skipping, as well as diurnal fluctuations of nutrient intake) shown in several studies may also contribute to differences in cardiometabolic health between chronotypes^6,13,16,28,29^. In the current study, the first and last eating occasions were analyzed, with the last food intake being significantly earlier in the morning subjects. Furthermore, it could be suggested that a late meal schedule may misalign metabolic functions controlled by tissue clocks with a central pacemaker controlled by the environmental light–dark cycle. This misalignment has been recognized as a cause of the higher incidence of metabolic disease in shift workers^30^ and has been confirmed in studies using simulated night shift work^31^, however, it still needs to be investigated in evening chronotypes. Thus, we suggest that metabolic disorders are less common for subjects with the morning chronotype due to a healthier diet and an earlier last eating occasion.

Modifiable lifestyle factors, such as physical activity and smoking, differ between chronotypes and may influence metabolic risk^18,20^. Morning types tend to be more physically active^6,7,18,20,32^, while evening types show a higher proportion of smokers^20,32^. In our cohort, morning chronotypes spent less time sitting, however, the proportion of current smokers was the same. Sleep parameters and circadian misalignment also contribute to adverse health outcomes in evening chronotypes. Evening chronotypes often experience poor sleep quality and sleep insufficiency^3,20,33^, but in other studies the effect of chronotype on sleep quality is inconsistent^7,34^. Moreover, studies have shown that sleep duration and sufficiency do not modify the association between chronotype and metabolic disorders^32,34,35^. In our study, sleep duration differed only on workdays, with shorter duration for evening types, while sleep quality was unaffected.

Circadian misalignment caused by work schedules is quantified using SJL, which reflects the discrepancy between biological and social clocks. Morning types in the present study had lower SJL, consistent with earlier findings^3,33^. Greater SJL has been previously associated with unhealthy lifestyles and poorer metabolic profiles^36–38^. Higher SJL was also linked to less adherence to the Mediterranean diet, increased likelihood of skipping breakfast and higher energy intake^36,38^. However, the relationship between SJL, diet, and obesity is unclear among different chronotype groups^39^. Therefore, the impact of SJL on health outcomes in evening types remains uncertain. Other measures of circadian discrepancy may be necessary to assess the circadian misalignment in these individuals.

This study has several limitations. Firstly, the cross-sectional design restricts our ability to establish causal relationships. Additionally, the assessment of individual chronotypes relied on a subjective questionnaire rather than objective measures such as dim light melatonin onset (DLMO). Nevertheless, previous research has demonstrated a significant correlation between the validated MEQ used in this study and DLMO^40^. It should be noted that the median MEQ score (58 points) used to categorize our sample falls on the borderline between morning and intermediate chronotypes. Thus, our findings primarily compare morning and intermediate MEQ types, challenging the assumption that the intermediate type is metabolically neutral, as suggested in previous studies that mainly focused on extreme chronotypes. Another limitation pertains to the self-reported nature of sleep-related variables, introducing the potential for participant bias. Additionally, the study is constrained by a small sample size. Future studies incorporating actigraphy or polysomnography can help overcome these limitations. Recognizing the multifaceted nature of estimating food consumption, we concur that various errors may arise not only from methodological considerations but also from factors such as differences in the actual nutrient content of foods and variations in individual responses to nutrients. In light of these complexities, our results regarding dietary intake associations should be interpreted with caution. Moreover, the present study and did not explore diurnal changes in dietary composition, warranting further investigation.

Despite these limitations, the present study has notable strengths. The inclusion of both middle-aged and elderly subjects with a wide range of metabolic phenotypes enhances the generalizability of our findings. The use of weighed 7-day food diaries for dietary intake analysis provides a more accurate assessment compared to methods such as the FFQ or 24-h recalls. Furthermore, energy-adjusted nutrient intake was employed for between-group comparisons and regression analysis, reducing external variation caused by individual metabolic rates.

In conclusion, the present data provide evidence for a different dietary pattern in morning chronotypes. The dietary composition observed in morning-type individuals, characterized by lower fat and animal protein intake and an earlier last eating occasion, may contribute to the associations between a higher chronotype score and a more favorable metabolic profile. Importantly, the association of morning chronotype with lower BMI, waist circumference, fasting triglycerides and glucose, and better overall metabolic health was independent of age and lifestyle differences. However, the underlying mechanisms of such effects remain to be elucidated. Longitudinal studies should investigate whether dietary and eating patterns mediate the metabolic effects of a specific chronotype. Additionally, interventional studies with different eating patterns considering meal timing and nutrient distribution are needed to clarify the mechanisms and causality of these associations.

## Material and methods

### Study population

This cross-sectional study was conducted from September 2020 to February 2022 in the urban area of Kyiv, Ukraine. The study included 142 Caucasian adult subjects consisting of outpatients of the D. F. Chebotarev State Institute of Gerontology and healthy volunteers. Ethical approval was obtained from the medical ethics committee of the D.F. Chebotarev State Institute of Gerontology and written informed consent was obtained from all subjects in accordance with the Declaration of Helsinki. All subjects underwent examinations, including blood sampling, anthropometric measurements, and the completion of specific questionnaires to assess health, medication intake, lifestyle, sleep behavior, chronotype (MEQ), and dietary assessment (based on 7-day weighed food records). The inclusion criteria were as follows: both males and females aged between 30 and 75 years; and absence of significant medical or psychiatric comorbidities. Exclusion criteria included a current diagnosis of a major illness (such as infectious disease, recent surgery, cancer, congestive heart failure, severe hepatic or renal diseases, mental illness), type 1 diabetes, thyroid disorders (hyperthyroidism, hypothyroidism, or thyroid hormone replacement therapy). Shift workers, patients with type 2 diabetes, and patients receiving drugs that influence glucose metabolism (e.g., metformin) were also excluded from the final sample. The final analytical sample comprised 110 subjects who met the inclusion and exclusion criteria.

### Study design

Subjects were recruited on an ongoing basis through the policlinic of the D. F. Chebotarev State Institute of Gerontology and advertisement in social media. All interested subjects were pre-screened by telephone, informed about the study protocol and then invited to the clinical center for the screening procedures. The screening was carried out by the trained staff of the D.F. Chebotarev State Institute of Gerontology. For the screening examination, the subjects appeared in the morning after 12 h of fasting. After the signing of the informed consent, the screening questionnaire was used to assess the general health status and to determine eligibility. After enrollment, subjects directly underwent a screening examination, including anthropometric measurements (i.e., height, weight, BMI, WC, blood pressure measurement, and taking of blood samples). Subjects then received lifestyle, sleep behavior, and chronotype questionnaires to be completed at home, a digital kitchen scale, and verbal and written instruction on how to keep weighed food records for 7 consecutive days. The food diaries were filled out manually and included the date, time, and weight of the food or drink consumed. On days 8–10 after the screening visit, subjects returned the completed questionnaires and food records to the clinic center. Participants had face-to-face contact with study personnel who checked for completeness and asked clarifying questions in case of inaccuracies.

### Chronotype and sleep assessment

Chronotype was assessed using the MEQ which includes 19 items about the subjective feeling of sleep and wakefulness during the day, and the preferred time to perform various tasks. MEQ scores range from 16 to 86^41^. The original MEQ distinguishes five chronotype categories, with higher scores indicating morning type—definitely morning type (70–86 points), moderately morning type (59–69 points), neither type (42–58 points), moderately evening type (31–41 points), and definitely evening type (16–30 points). For the purposes of the study, the number of groups can be reduced to three chronotypes—morning type (59–86 points), neither (intermediate) type (42–58 points), and evening type (16–41 points)^14,34^.

Sleep timing was obtained from the Munich Chronotype Questionnaire, which includes variables such as sleep onset on workdays, sleep onset on free days; sleep duration on workdays; sleep duration on free days; the midpoint of sleep on free days corrected for workweek sleep debt (MSFsc), and SJL^3^. It is worth noting that MSFsc can also be used to chronotype subjects with an earlier mid-sleep time corresponding to the earlier type^3^. The limitation of MSFsc is that it can only be calculated for individuals who do not use an alarm clock to wake up on free days. We used MSFsc as a temporal parameter of the sleep–wake cycle for the between-group comparison. SJL was calculated as the absolute difference between midpoints of sleep on days off and midpoints of sleep on workdays^42^.

Subjective sleep quality was assessed using the PSQI with a total score ranging from 0 to 21^43^. A PSQI total score > 5 was considered to indicate poor sleep quality.

### Anthropometrics and blood pressure

Anthropometric measurements included body weight, height, and WC. Body weight was measured to the nearest 0.1 kg and height to the nearest 0.1 cm using calibrated scales and a stadiometer. BMI was calculated as weight (kg) divided by height (m) squared. WC was measured without clothing, directly on the skin. WC was measured at the midpoint between the lower rib margin and the iliac crest. Blood pressure was measured three times after 10 min of rest in a sitting position. All staff were trained to perform the measurements.

### Dietary assessment

Dietary intake was assessed using 7-day weighed food records. All subjects were provided with a digital kitchen scale, verbal and written instructions on how to weigh and record all foods and beverages consumed for a period of 7 consecutive days. All records were then checked for completeness by the study personnel. We defined a food diary as complete if it contained a completed entry for each meal or confirmation of a missed meal for each day. The completion rate in our sample was 94%. Dietary data obtained from the food diaries were analyzed by a trained dietitian using TRP-D02 software version 2020 (Viria, Kyiv, Ukraine)^44^ to obtain daily nutrient intakes. Consumption of all nutrients was adjusted for energy using the residual method^45^. For the purpose of this study, foods were categorized into 9 groups: total dairy; total meat and eggs; fish and seafood; starchy foods; vegetables and legumes; berries and fruits; nuts, seeds, and peanuts; confectionery and sweet bakery products; honey and sugar. The total dairy group included all fat varieties of milk, kefir, and yogurt, and various types of cheese (hard, soft, sour cheese, and curd). The total meat and eggs group included red meat, poultry, processed meat, and eggs. The group of starchy foods consisted of cereals and pasta in dry weight, bread, and potatoes. Confectionery (candy, halvah, chocolate, ice cream) was grouped together with sweet bakery products (cookies, cakes, biscuits). Foods were not adjusted for total energy by residual method because of the high incidence of zero consumption for some types of food. The term "eating occasion" was defined as any instance of caloric consumption equal to or greater than 50 kilocalories. The average times of the first and last eating occasions were determined from the 7-day food diaries. The fasting window was then calculated as the number of hours between the last and first eating occasions based on these records. Subjects were considered to be breakfast skippers if they did not eat any solid food between 4:00 and 12:00 a.m. on at least 4 days per week. Meal regularity was obtained from the question; "Do you follow a regular eating pattern? Yes/No".

### Assessment of physical activity and smoking

Physical activity was assessed using a brief self-administered version of the IPAQ by calculating the total MET minutes per week^46^. To obtain total MET minutes of physical activity, MET minutes of walking, moderate and vigorous activity were summed. Subjects were defined as "current smokers" if they reported smoking at least one cigarette per day and as "non-smokers" if they reported not or occasionally smoking.

### Biochemical analysis and metabolic syndrome scoring

Venous blood samples were collected after an overnight fast in the morning. Blood samples were collected into a 4 mL tube with K2EDTA (Vacutest ref 135300; Vacutest KIMA, Arzergrande, Italy) for screening complete blood count and two 5 mL tubes with clot activator and serum separator gel (Vacutest ref 10176; Vacutest KIMA, Arzergrande, Italy) for serum. Blood samples for serum were centrifuged (3000 rpm, 10 min) and serum total cholesterol, LDL-C, and HDL-C, as well as triglycerides, were estimated using commercially available test kits (BioSystems S.A., Barcelona, Spain) in a semi-automatic biochemistry analyzer BTS-350 (BioSystems S.A., Barcelona, Spain). Serum glucose was estimated by the glucose oxidase method using a test kit (Human, Wiesbaden, Germany).

Serum samples for insulin determination were stored at − 70 °C until analysis. Insulin was determined in duplicates using commercially available kits (DRG Instruments GmbH, Marburg, Germany) based on the double-antibody sandwich ELISA method. The HOMA-IR (fasting insulin concentration (μIU/mL) * fasting glucose concentration (mmol/L)/22.5) was used to assess insulin resistance. To assess metabolic risk factors, we used the AHA/NHLBI (ATP III) criteria for MetS (Joint Interim Statement for harmonizing the MetS), which include: (a) WC ≥ 88 cm in females or ≥ 102 cm in males); (b) fasting triglycerides ≥ 1.7 mmol/L or specific treatment for this lipid abnormality; (c) fasting HDL-C levels of < 1.29 mmol/L in females and < 1.03 mmol/L in males, or specific treatment for this lipid abnormality; (d) blood pressure of ≥ 130/85 mm Hg or treatment for a previous diagnosis of hypertension; and (e) hyperglycemia with fasting glycemia ≥ 5.6 mmol/L^47^. MetS scores were calculated by summing the presence of each MetS component (WC, fasting triglycerides, HDL-C, blood pressure, and glucose)^47^. In the present study, being metabolically healthy was defined as the absence of any of the aforementioned MetS criteria.

### Statistics

The normality of data was assessed using the Kolmogorov–Smirnov test. Continuous data are presented as mean with standard deviation (SD) or median with interquartile range (IQR), depending on the normality distribution. Variables of metabolic parameters and nutrient intake that did not meet the normality assumption were log-transformed. Differences between means were compared by t-test and between medians by—Mann–Whitney U-test. Categorical variables are presented as numbers and percentages, and differences were assessed by cross-tabulation using Fisher's exact test.

To assess the relationships between food group intake and MEQ score, Spearman’s correlation was used due to the skewness of most food group intake data. The associations of nutrients and metabolic risk factors with chronotype were analyzed using linear regression with MEQ score as a continuous variable. To examine the relationship between chronotype and high blood pressure, the logistic regression model was constructed because of the intake of antihypertensive medication by some study subjects (blood pressure < 130/85 mmHg = 0; blood pressure ≥ 130/85 or antihypertensive medication use = 1). The association between overall metabolic health (absence of MetS criteria = 0, presence of ≥ 1 MetS criteria = 1) and chronotype score was assessed with logistic regression models. The odds of being metabolically unhealthy (meeting ≥ 1 MetS MetS criteria) were assessed for each 10-point change in MEQ scores. EZR statistics software version 1.55 (The R Foundation for Statistical Computing, Vienna, Austria) was used for data comparison between chronotype groups and regression analysis^48^. Energy adjustment of nutrients was performed in IBM SPSS Statistics software version 28 (IBM Corp., Chicago, IL, USA). A p-value of less than 0.05 was considered statistically significant. All plots were built in R Statistical Software version 4.2.3 (The R Foundation for Statistical Computing, Vienna, Austria)^49^ using jtools^50^ and broom.mixed^51^ packages.

### Supplementary Information

Supplementary Information.

## Data Availability

The datasets used and/or analyzed during the current study are available from the corresponding author on reasonable request.
