# Supplementary material for: Association of diet, lifestyle, and chronotype with metabolic health in Ukrainian adults: a cross-sectional study
Source: Sci Rep. 2024 Mar 1;14:5143. doi: 10.1038/s41598-024-55715-0 (PMC10907368; doi:10.1038/s41598-024-55715-0)
Supplement: Supplementary file 1 — Supplementary Information. [file 41598_2024_55715_MOESM1_ESM.pdf]

Association of Diet, Lifestyle, and Chronotype with Metabolic Health in Ukrainian Adults: A cross-sectional study

Mariana Romanenko[1,2*], Julius Schuster[2], Liubov Piven[1], Liudmyla Synieok[1], Tetyana Dubiley[1], Liudmyla Bogomaz[1], Andreas Hahn[2], Mattea Müller[2]

[1]D.F. Chebotarev State Institute of Gerontology NAMS of Ukraine, Vyshgorodska str. 67, 04114 Kyiv, Ukraine

[2] Institute of Food Science and Human Nutrition, Leibniz University Hannover, am Kleinen Felde 30, 30167 Hannover, Germany

mr@geront.kiev.ua

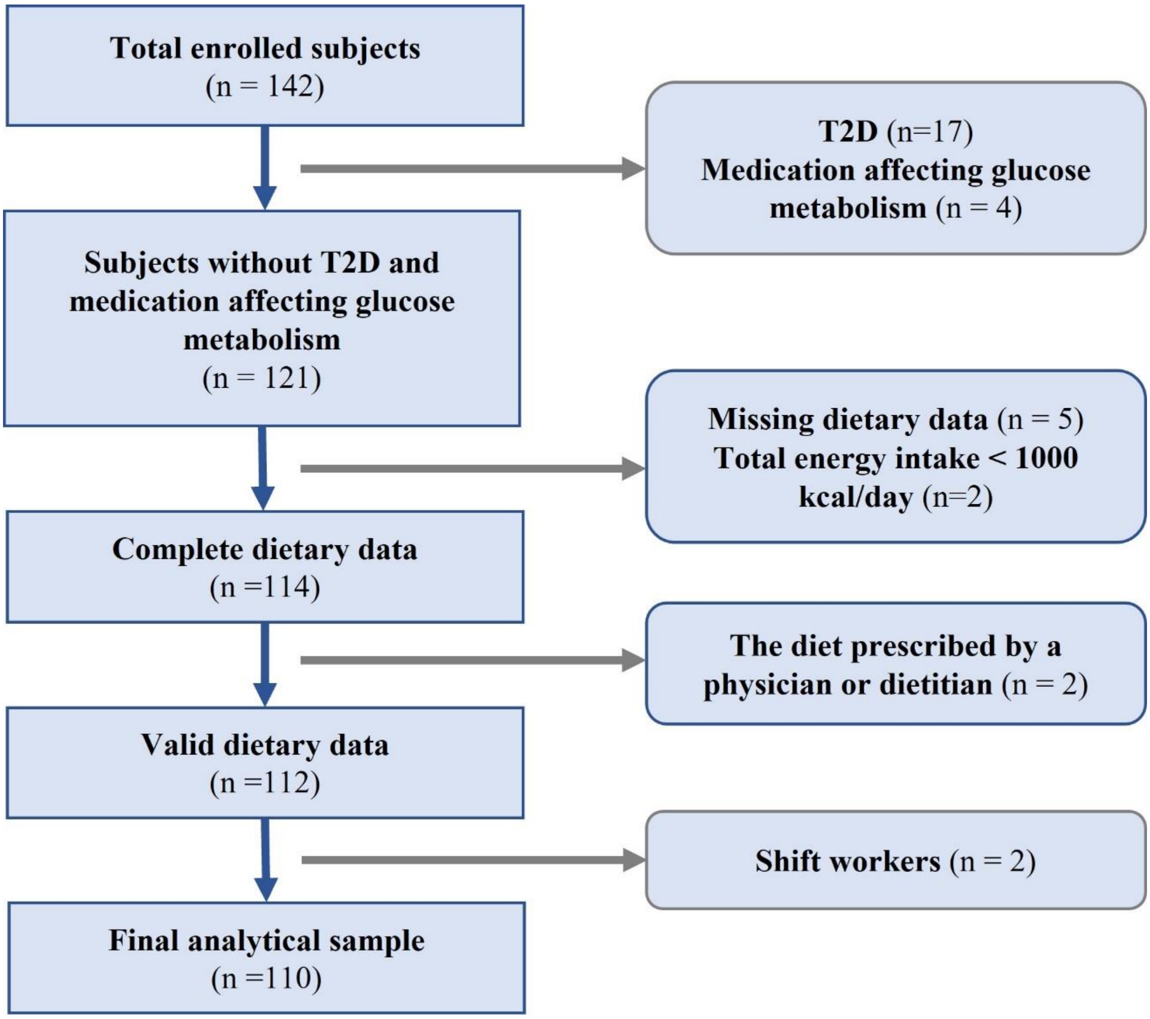

Supplementary Figure S1. Study flowchart. Abbreviation: T2D, Type 2 Diabetes.

Supplementary Table S1. Association of chronotype (MEQ score) with energy-adjusted nutrient intake[1].

	**std. B (SE)**	**std. 95% CI**	**P**	**R^2 adjusted**
Total protein, g/day	-0.17 (0.10)	-0.38 – 0.03	0.098	0.049
% E total protein	-0.15 (0.10)	-0.35 – 0.06	0.155	0.034
Animal protein, g/day	-0.30 (0.10)	-0.50 – -0.10	**0.004**	0.088
Plant protein, g/day	0.28 (0.10)	0.08 – 0.48	**0.007***	0.055
Total fat, g/day	-0.27 (0.10)	-0.46 – -0.07	**0.008**	0.134
% E total fat	-0.27 (0.10)	-0.47 – -0.08	**0.007**	0.129
Animal fat, g/day	-0.32 (0.10)	-0.52 – -0.12	**0.002**	0.089
Plant fat, g/day	0.08 (0.10)	-0.12 – 0.29	0.418	0.057
Carbohydrate, g/day	0.27 (0.10)	0.07 – 0.46	**0.008**	0.137
% E carbohydrate	0.28 (0.10)	0.08 – 0.47	**0.005**	0.148
Sugar, g/day	0.07 (0.10)	-0.14 – 0.28	0.497	0.009
Starch, g/day	0.19 (0.10)	-0.01 – 0.39	0.063	0.074
Fiber, g/day	0.19 (0.10)	-0.01 – 0.40	0.059	0.065
Total energy, kcal/day	-0.06 (0.09)	-0.25 – 0.12	0.493	0.243

The association is calculated for every 10-point change in the MEQ score. Models controlled for age, sex, and physical activity.

[1] – Nutrient intake was energy-adjusted by the residual method; macronutrients are additionally presented as a % of energy.

* – overall model p-value is not significant.

Abbreviations: MEQ, Morningness-Eveningness Questionnaire; B, beta coefficient; SE, standard error; CI, confidence interval; E – energy.

Supplementary Table S2. Spearman's correlation and partial correlation between chronotype (MEQ score) and food group intake.

	Spearman r correlation coefficients	P-value	Spearman r partial correlation coefficients[2]	P-value
Total dairy, g/day	-0.09	0.356	-0.12	0.247
Milk/Kefir/Yogurt, g/day	-0.06	0.569	-0.08	0.421
Cheese, g/day	**-0.21**	**0.032**	**-0.21**	**0.042**
Total meat and eggs, g/day	**-0.23**	**0.022**	-0.17	0.102
Red meat, g/day	-0.16	0.100	-0.13	0.194
Poultry, g/day	0.04	0.698	0.16	0.111
Processed meat, g/day	-0.17	0.091	**-0.20**	**0.047**
Eggs, g/day	**-0.23**	**0.021**	**-0.21**	**0.041**
Fish and seafood, g/day	-0.04	0.670	0.002	0.980
Starchy food, g/day	0.13	0.188	0.12	0.250
Cereals and pasta, g/day of dry weight	0.10	0.306	0.12	0.237
Bread, g/day	0.16	0.122	0.10	0.309
Potato, g/day	0.07	0.471	0.07	0.512
Vegetables and legumes, g/day	0.04	0.710	0.067	0.509
Vegetables, g/day	0.03	0.791	0.05	0.661
Legumes, g/day	0.13	0.195	0.19	0.060
Berries and fruit, g/day	-0.04	0.714	-0.01	0.917
Nuts, seeds, and peanuts, g/day	0.01	0.915	0.11	0.303
Confectionery and sweet bakery products, g/day	0.01	0.906	0.08	0.449
Honey and sugar, g/day	-0.02	0.856	-0.03	0.771

[2]Partial correlation coefficients controlled for age, sex, physical activity, and total energy intake.
Abbreviations: MEQ, Morningness-Eveningness Questionnaire.

Supplementary Table S3. Association of chronotype (MEQ score) with metabolic parameters

	Model 1				Model 2			
	**std. B (SE)**	**std. 95% CI**	**P**	**R^2 adjusted**	**std. B (SE)**	**std. 95% CI**	**P**	**R^2 adjusted**
BMI, kg/m^2	-0.21 (0.10)	-0.41 – -0.02	**0.029**	0.078				
Waist,cm	-0.20 (0.09)	-0.39 – -0.02	**0.032**	0.136				
WHR	-0.004 (0.08)	-0.16 – 0.16	0.958	0.360				
Total cholesterol, mmol/L	0.12 (0.10)	-0.07 – 0.32	0.211	0.038	0.14 (0.10)	-0.06 – 0.34	0.179	0.032
HDL-C, mmol/L	0.28 (0.10)	0.09 – 0.47	**0.005**	0.084	0.23 (0.10)	0.04 – 0.42	**0.020**	0.134
LDL-C, mmol/L	0.12 (0.10)	-0.07 – 0.32	0.218	0.015	0.14 (0.10)	-0.07 – 0.34	0.187	0.009
Triglycerides, mmol/L	-0.24 (0.09)	-0.4 – -0.06	**0.009**	0.185	-0.18 (0.09)	-0.36 – -0.00	**0.045**	0.250
Glucose, mmol/L	-0.27 (0.10)	-0.46 – -0.07	**0.007**	0.066	-0.26 (0.10)	-0.46 – -0.06	**0.010**	0.057
Insulin, µIU/ml	-0.16 (0.10)	-0.35 – 0.03	0.101	0.105	-0.08 (0.09)	-0.26 – 0.10	0.399	0.231
HOMA – IR, points	-0.20 (0.10)	-0.39 – -0.01	**0.037**	0.102	-0.12 (0.09)	-0.30 – 0.06	0.183	0.224

The association is calculated for every 10-point change in the MEQ score. Model 1 controlled for age, sex, and physical activity. Model 2 – model 1 plus controlled for BMI.

Abbreviations: B, beta-coefficient; SE, standard error; CI, confidence interval; MEQ, Morningness-Eveningness Questionnaire; BMI, body mass index; WHR, waist-to-hip ratio; HDL-C, high-density lipoprotein cholesterol; LDL-C, low-density lipoprotein cholesterol; HOMA-IR, homeostatic model assessment of insulin resistance.
